# MicroRNA-200c Modulates the Expression of MUC4 and MUC16 by Directly Targeting Their Coding Sequences in Human Pancreatic Cancer

**DOI:** 10.1371/journal.pone.0073356

**Published:** 2013-10-25

**Authors:** Prakash Radhakrishnan, Ashley M. Mohr, Paul M. Grandgenett, Maria M. Steele, Surinder K. Batra, Michael A. Hollingsworth

**Affiliations:** 1 Eppley Institute for Research in Cancer and Allied Diseases, University of Nebraska Medical Center, Omaha, Nebraska, United States of America; 2 Department of Biochemistry and Molecular Biology, University of Nebraska Medical Center, Omaha, Nebraska, United States of America; University of Saarland Medical School, Germany

## Abstract

Transmembrane mucins, MUC4 and MUC16 are associated with tumor progression and metastatic potential in human pancreatic adenocarcinoma. We discovered that miR-200c interacts with specific sequences within the coding sequence of MUC4 and MUC16 mRNAs, and evaluated the regulatory nature of this association. Pancreatic cancer cell lines S2.028 and T3M-4 transfected with miR-200c showed a 4.18 and 8.50 fold down regulation of MUC4 mRNA, and 4.68 and 4.82 fold down regulation of MUC16 mRNA compared to mock-transfected cells, respectively. A significant reduction of glycoprotein expression was also observed. These results indicate that miR-200c overexpression regulates MUC4 and MUC16 mucins in pancreatic cancer cells by directly targeting the mRNA coding sequence of each, resulting in reduced levels of MUC4 and MUC16 mRNA and protein. These data suggest that, in addition to regulating proteins that modulate EMT, miR-200c influences expression of cell surface mucins in pancreatic cancer.

## Introduction

MicroRNAs (miRNAs) are small (~20-22 nucleotides) non-coding RNAs that regulate gene expression by interacting with either the 3’ untranslated region (3’ UTR) or coding region of target mRNAs. Expression of targeted gene products are affected by miRNA induced mRNA degradation or inhibition of translation [1]. Altered expression of miRNAs in cancer results in tumor promoting and tumor suppressing functions. For example, miR-155, miR-17-5p and miR-21 have known oncogenic activities [2,3], whereas, miR-15a, miR-16-1, let-7 and miR-145 function as tumor suppressors [4-9]. It has been shown that miR-200c is differentially expressed in pancreatic cancer, where high expression was associated with better survival and low expression is associated with worse survival [10]. Overexpression of miR-200c inhibits cancer cell invasion by modulating factors important in EMT [10]. Ectopic expression of miR-200c induces higher levels of E-cadherin in breast and pancreatic cancer cells through the direct targeting of transcription factor 8 (TCF8/ ZEB1), a negative regulator of E-cadherin [11-14]. Therefore, cells with high levels of miR-200c have a more epithelial and less mesenchymal phenotype that may impact metastasis. Recently, overexpression of miR-200c was shown to inhibit melanoma tumor progression and drug resistance [15].

Aberrant expression of mucin glycoproteins is associated with pancreatic cancer progression to metastasis. The transmembrane mucins, MUC4 and MUC16, are aberrantly overexpressed in many adenocarcinomas, including human pancreatic cancer [16-18]. The MUC4 mucin is a large glycoprotein expressed by epithelial cells in a variety of tissues. MUC4 is not expressed in the normal pancreas, but is aberrantly expressed in a high percentage of premalignant and malignant pancreatic lesions [16,19]. MUC4 promotes cancer growth and metastasis in part through interaction with the HER2 oncoprotein [20,21]. MUC16 (CA125) is a high molecular weight mucin glycoprotein normally expressed in ocular surface, respiratory tract and reproductive organs containing epithelial cells [22]. CA125, an epitope on MUC16, is used as a tumor marker for detection of ovarian cancer in sera of patients [23] and MUC16 influences ovarian cancer growth and metastasis [24]. Increased expression of MUC16 is also observed in human pancreatic cancer [17,18], and we have recently shown that there is increased expression in metastatic lesions of pancreatic cancer patients [25].

We report here that miR-200c interacts with specific sequences within the coding sequence of MUC4 and MUC16 mRNAs and regulates their levels of expression. Higher expression of transmembrane mucins and loss of miR-200c are highly associated with metastatic characteristics of cancer cells.

## Materials and Methods

### Cell lines and culture

Human pancreatic cancer cells Capan-1 (American Type Culture Collection, ATCC), S2.007, S2.013, S2.020, S2.028 [26], HPAF (Generous gift from R.S. Metzgar, Department of Microbiology and Immunology, Duke University Medical Center, Durham. North Carolina), and T3M-4 (Kind gift from Tetsuro Okabe, University of Tokyo, Japan) were grown in Dulbecco’s Modified Eagle’s Medium (DMEM) (Hyclone, Logan, UT, USA), supplemented with 10% Fetal bovine serum (Valley Biomedical Inc., Winchester, VA, USA), 100 µg/mL streptomycin and 100 units/mL penicillin (Mediatech Inc., Manassas, VA, USA) and incubated at 37°C with 5% CO_2_ in a humidified incubator.

### microRNA isolation and Real time-PCR (RT-PCR) analysis of miR-200c

miRNA were extracted from pancreatic cancer cells using mirVana miRNA isolation kit (Ambion, Carlsbad, CA, USA). Expression of miR-200c was quantified using TaqMan MicroRNA assay kit (ABI, Carlsbad, CA, USA) according to the manufacturer’s protocol.

### miRNA-200c overexpression in S2.028 and T3M-4 pancreatic cancer cell lines

Oligonucleotides of the primary transcript of miR-200c were cloned into p*Silencer* 4.1-CMV plasmid (Ambion, Carlsbad, CA, USA) (Table S1) to establish a stable expression vector. Cells were seeded at 75 x10^3^ cells per well (12-well plate) the day prior to transfection, and 1 µg plasmid was transfected into the cells the following day using Lipofectamine LTX with PLUS reagent (Invitrogen life technologies, Carlsbad, CA, USA) according to the manufacturer’s protocol. Cells were placed under selection with G418 (200µg/ml) 48 hours after transfection. Clones were subsequently picked and analyzed for miR-200c expression in the S2.028 cell line (clone 7), while a polyclonal selected population was utilized for the T3M-4 cell line. Cells transfected with scrambled sequence containing vector from the kit (Ambion, Carlsbad, CA, USA) served as a negative control. Expression of miR-200c was quantified using TaqMan MicroRNA assay kit (ABI, Carlsbad, CA, USA) according to the manufacturer’s instructions. U6 rRNA was used as an internal control. The fold-change in expression was calculated by using the 2^-ΔΔCt^ method [27].

### Methylene Blue Cell Proliferation Assay

Cell proliferation was assayed using a methylene blue cell dye as previously described [28]. S2.028 and T3M-4 control and miR-200c expressing cells were counted and plated at 2,000 cells per well in a 96-well plate and 200 µl total volume, eight replicates for each time frame. At each time point (24, 48, 72 and 96 hours) media were removed and cells were washed once with 150 µl Dulbecco’s PBS and fixed with 150 µL 10% formalin for 30 minutes at room temperature. Formalin was removed and cells were stained for 2 hours with 80 µL of 1% Methylene Blue (Sigma-Aldrich, Saint Louis, MO, USA) diluted with 0.01 M borate buffer, pH 8.5. Cells were washed with 150 µL of 0.01 borate buffer, pH 8.5, four times. Methylene blue was extracted with 100 µL of 0.1 N HCl/Ethanol (1:1) and incubated for 30 minutes at room temperature. Absorbance at 650 nm was determined by spectrophotometric analysis. Two Way ANOVA was used to analyze the statistical difference between the groups. A p value of less than 0.05 was considered statistically significant.

### Real-time PCR (RT PCR) analysis of mucin expression

Total RNA was isolated from S2.028 and T3M-4 cells using TRI Reagent (Molecular Research Center, Cincinnati, OH, USA) according to the manufacturer’s instructions. cDNA was synthesized by using Verso cDNA kit (Thermo, Fisher Scientific, Waltham, MA, USA). Analysis of MUC1, MUC4, MUC16 and GAPDH mRNA levels were performed with SYBR Green PCR master mix (ABI, Carlsbad, CA, USA). The following primers were used for RT-PCR: MUC1, F-5-CTGCTCCTCACAGTGCTTACAGTTG-3; R-5- TGAACCGGGGCTGTGG CTGG-3; MUC4, F-5-GCCCAAGCTACAGTGTGACTCA-3; R-5-ATGGTGCCGTTGTAATT TGTTGT-3; MUC16, F-5-ACATCAACTCCTGCCTTCCCAGAA-3; R-5-ACCAGTGGGCAT TCCAGAAAGAGA-3; GAPDH, F-5-TCGACAGTCAGCCGCATCTTCTTT-3; R-5-ACCAA ATCCGTTGACTCCGACCTT-3. Each experiment was carried out in triplicate. Differences in mucin gene expression, expressed as fold-changes, were calculated using the 2^-ΔΔCt^ method using GAPDH as internal control.

### Immunoblot analysis of Mucins

SDS-Agarose gel electrophoresis was used to analyze mucin expression as described previously [29]. Cells expressing miR-200c or a scrambled control were harvested and protein was extracted using NP-40 cell lysis buffer (150mM NaCl, 50mM Tris-HCl, 1% NP-40, pH8.0) supplemented with protease inhibitors (Promega, Madison, WI, USA). Cell lysates (50-100 µg proteins) were resolved on SDS (0.1%) - Agarose (2%) gel electrophoresis and transferred to PVDF membrane. Membranes were blocked in 5% non-fat dry milk powder in Tris-buffered saline (150mM NaCl, 50mM Tris-HCl) with 0.1% Tween 20 pH 7.4. The membranes were incubated with the following primary antibodies: MUC1 (AR20.5-Mouse IgG, Quest PharmaTech Inc, Edmonton, Alberta, CA), MUC4 (8G7- Mouse IgG, kind gift from Dr. Surinder K Batra, Department of Biochemistry and Molecular biology, UNMC), MUC16 (AR9.6R333 Mouse IgG, Quest PharmaTech Inc, Edmonton, Alberta, CA) and α-tubulin (Mouse IgG, Developmental studies hybridoma bank, Iowa, USA) (1:1000 dilutions with 5% non-fat dry milk powder in TBS-T), overnight at room temperature. The membranes were subsequently washed (3×10 min) with TBS-T at room temperature and probed with 1:5000 diluted Goat-anti mouse HRP conjugated secondary antibody (Jackson ImmunoResearch, West Grove, PA, USA) for 1 h at room temperature and washed 3×10 min with TBS-T. The signal was detected with Super signal^®^ West Pico Chemiluminescent substrate (Thermo scientific, Rockford, IL, USA). A densitometric quantification of MUC4 and MUC16 protein bands were carried out using the ImageJ program (NIH). The fold change in protein band intensity (percent arbitrary units) was calculated by dividing the protein density of miR-200c expressing cells by the protein density of vector control cells. Detection of alpha tubulin was used as a loading control.

### Vector constructs

ORF regions of MUC16 and MUC4 mRNA that were predicted to interact with miR-200c were synthesized and inserted into pMIR-REPORT^TM^ miRNA Expression Reporter Vector system (ABI, Carlsbad, CA, USA) using the SpeI and HindIII restriction sites (New England BioLabs, Ipswich, MA, USA). Wild type and mutant MUC4 and MUC16/miR-200c interactions sequences were synthesized and used in this study (Table S1). Mutations within potential miR-200c binding sites were generated by nucleotide replacement of wild type sequence to inhibit miR-200c binding. The insertion and orientation of the fragment were confirmed by sequence analysis. The plasmids were designated pMIR-REPORT^TM^-wild (MUC16 and MUC4 wt) and pMIR-REPORT^TM^-mutant (MUC16 and MUC4 mt) (Table S1).

### Transfection and Luciferase assays

Pancreatic cancer (S2.028 and T3M-4) cells were cultured in a 6 well plate and transiently transfected at 60-80% confluence with the plasmids indicated above using the Lipofectamine^TM^ 2000 reagent (Invitrogen life technologies, Carlsbad, CA, USA) as per the manufacturer’s instructions. Luciferase activity was measured by using Luciferase Reporter Assay systems (Promega, Madison, WI, USA), 48 h after transfection. Luciferase assays were carried out according to the manufacturer’s instructions on a 96 well plate reader (Polar Star Optima Microplate Reader, Offenburg, Germany). All values were presented as Mean ± SEM of relative luciferase units (RLU) from three independent experiments performed in triplicates. An unpaired t-test (two-tailed) was performed for statistical analysis using the GraphPad PRISM® Version 5.0 program. Differences with a p value < 0.05 were considered statistically significant.

## Results

### miR-200c: expression profiling and overexpression in pancreatic cancer cell lines

**Figure 1:**
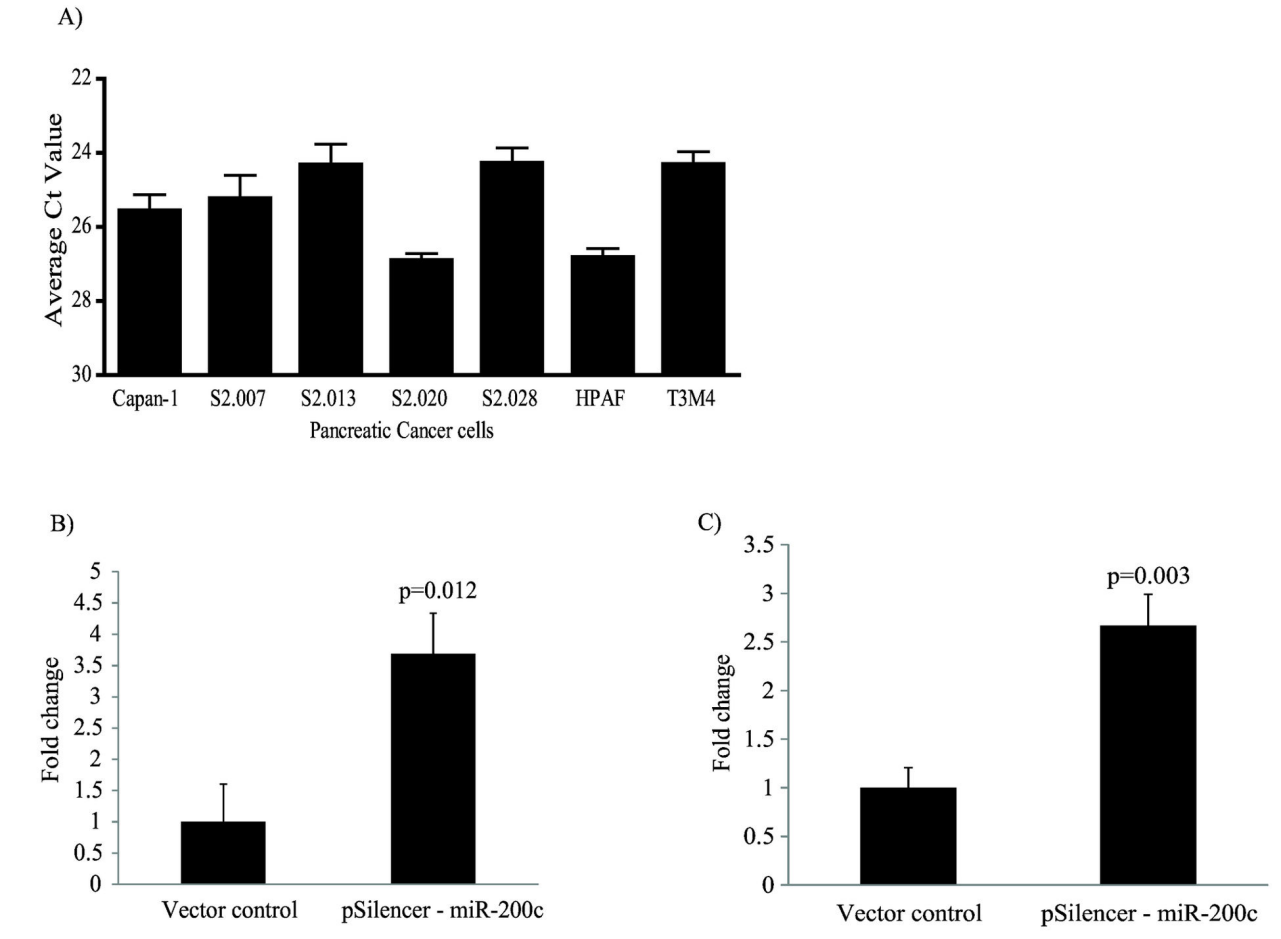
Quantitative analysis of miR-200c expression in human pancreatic cancer cell lines and ectopic expression of miR-200c in S2.028 and T3M-4 cells. (A) The expression of miR-200c in seven pancreatic cancer cells were determined by Real-time PCR. Each sample was run in quadruplicate and error bars represent SD. S2.028 (B) and T3M-4 cells (C) stably expressing the primary transcript of miR-200c were evaluated for miR-200c expression by Real-time PCR. Each measurement was carried out in triplicate. These values were normalized with internal control U6 rRNA. The fold increase in transcript levels over vector control is expressed as Mean ± S.D. The p value was determined by using the Student’s t-test. Differences with a p value < 0.05 were considered statistically significant.

We investigated the expression of miR-200c in 7 pancreatic cancer cell lines by quantitative real-time RT-PCR. As shown in Figure 1A, 5 pancreatic cancer cell lines, including Capan-1, S2.007, S2.013, S2.028, and T3M-4, express higher levels of miR-200c than S2.020 and HPAF cells.

The mature sequence of miR-200c was synthesized and cloned into an expression vector as described above (p*Silencer* 4.1-CMV-miR-200c), and stably expressed in S2.028 cells and T3M-4. Quantitative RT-PCR analysis of miR-200c expression in S2.028-miR-200c cells showed a 3.68 fold increase and T3M-4-miR-200c cells showed a 2.6 fold increase in mature miR-200c levels compared to vector control transfected cells (Figure 1B and 1C). We confirmed that the recombinant mature form of miR-200c was active by observing downregulation in the expression of ZEB1, a known target for miR-200c, in both S2.028 and T3M-4 cells (Figure S1).

We also examined the changes in the cell proliferation properties of miR-200c expressing S2.028 and T3M-4 pancreatic cancer cells. A significant reduction in growth rate of S2.028 miR-200c was observed at 72 and 96 hours (***p<0.0001) when compared to S2.028 vector control cells (Figure S2A). However, no change was observed when T3M-4 miR-200c was compared to vector control cells at any time point tested (Figure S2B).

### MUC4 and MUC16 expression is suppressed by miR-200c

**Figure 2:**
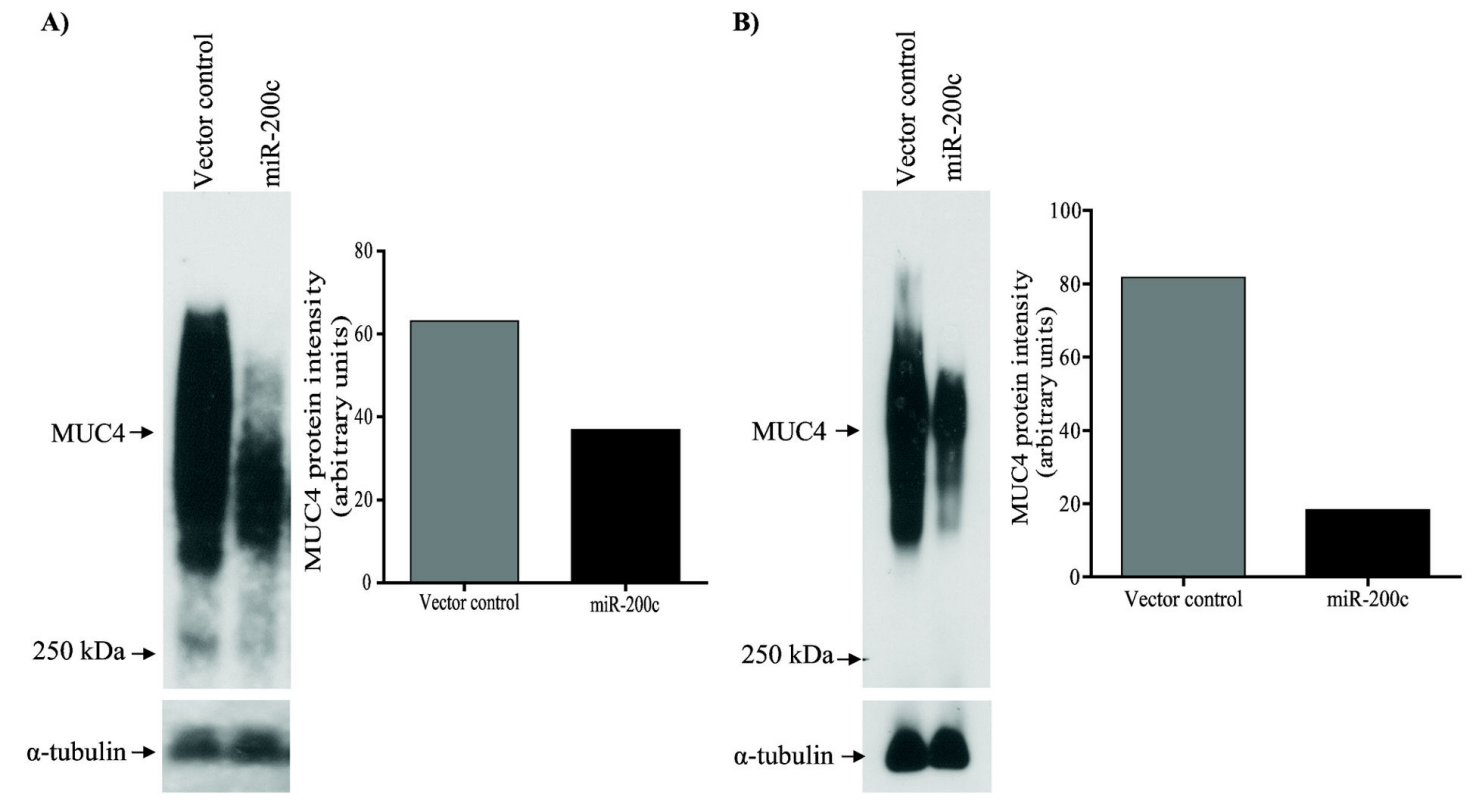
miR-200c targets MUC4. Lysates from (A) S2.028 and (B) T3M-4-miR-200c and respective vector control transfected cells were separated on SDS-Agarose gel electrophoresis, subjected to western blot using an anti-MUC4 antibody (left panels) and signals were quantified by densitometry and analyzed using the ImageJ program (right panels). The miR-200c expressing cells showed a significant reduction of MUC4 protein compared to control cells in (A) S2.028 and (B) T3M-4 cells. Detection of alpha tubulin was used as a loading control.

**Figure 3:**
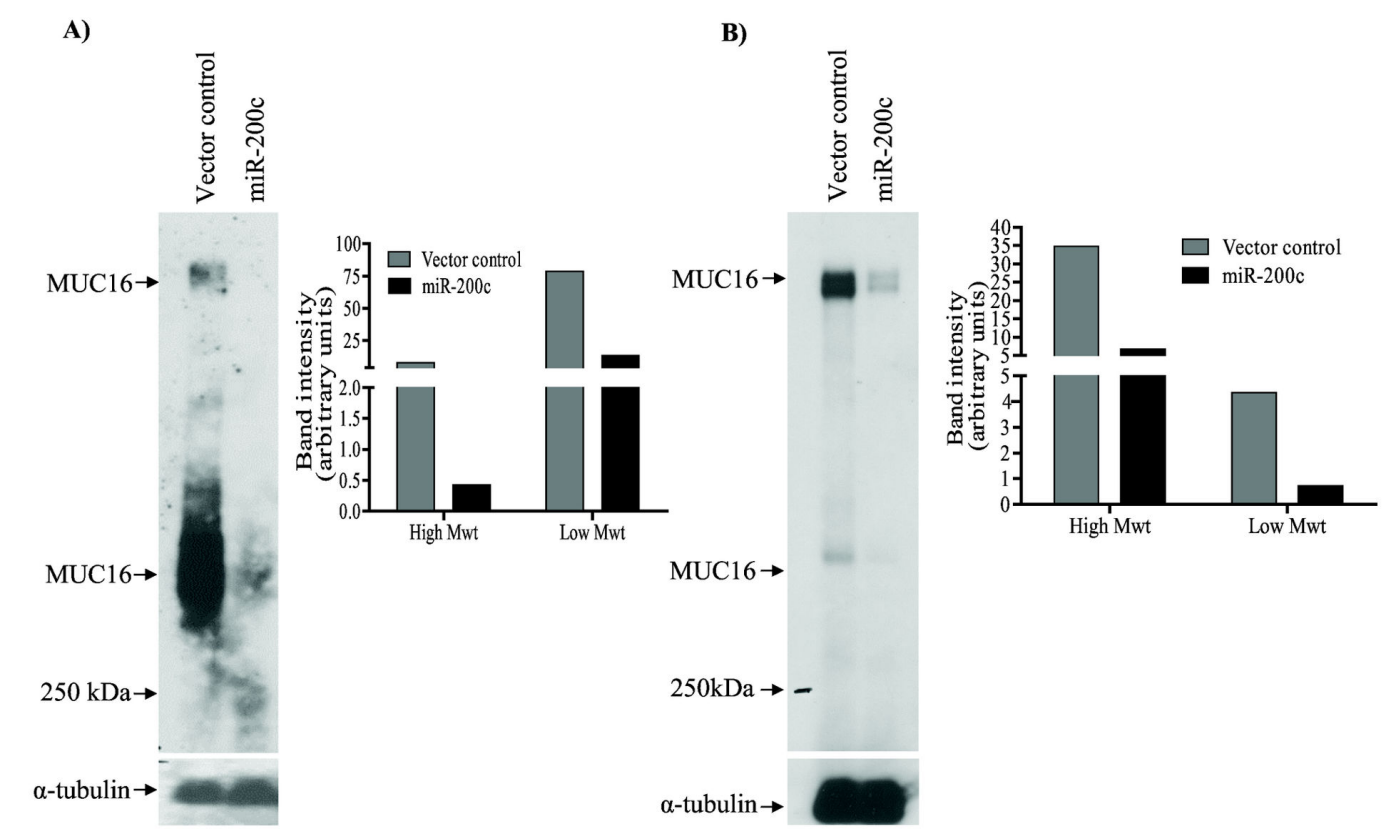
microRNA-200c targets MUC16. (A) S2.028 and (B) T3M-4-miR200c and respective vector control transfected cell lysates were separated by SDS-Agarose gel electrophoresis and subjected to western blot using an anti-MUC16 antibody (left panels). Band intensity was quantified by densitometry and analyzed using the imageJ program (right panels). Significant reductions of both high and low molecular weight MUC16 protein isoforms were observed in miR-200c expressing S2.028 (A) and T3M-4 cells (B) than compared to vector control cells. α-tubulin was used as a loading control.

We assessed the ability of miR-200c to regulate membrane bound mucins by expressing miR-200c (p*Silencer* 4.1-CMV-miR-200c) in pancreatic cancer cell lines S2.028 and T3M-4. There was a dramatic reduction of MUC4 protein in S2.028-miR200c (1.7 fold) and T3M-4-miR-200c (4.46 fold) cells relative to control cells (Figure 2A and 2B). Similarly, there was a significant reduction in MUC16 protein (high and low molecular weight isoforms) in S2.028-miR200c (18.1 and 5.9 fold respectively) and T3M-4-miR-200c (5.2 and 6.1 fold respectively) cells as compared to control cells (Figure 3A and 3B).

### miR-200c targets transcriptionally membrane bound mucins MUC4 and MUC16

**Figure 4:**
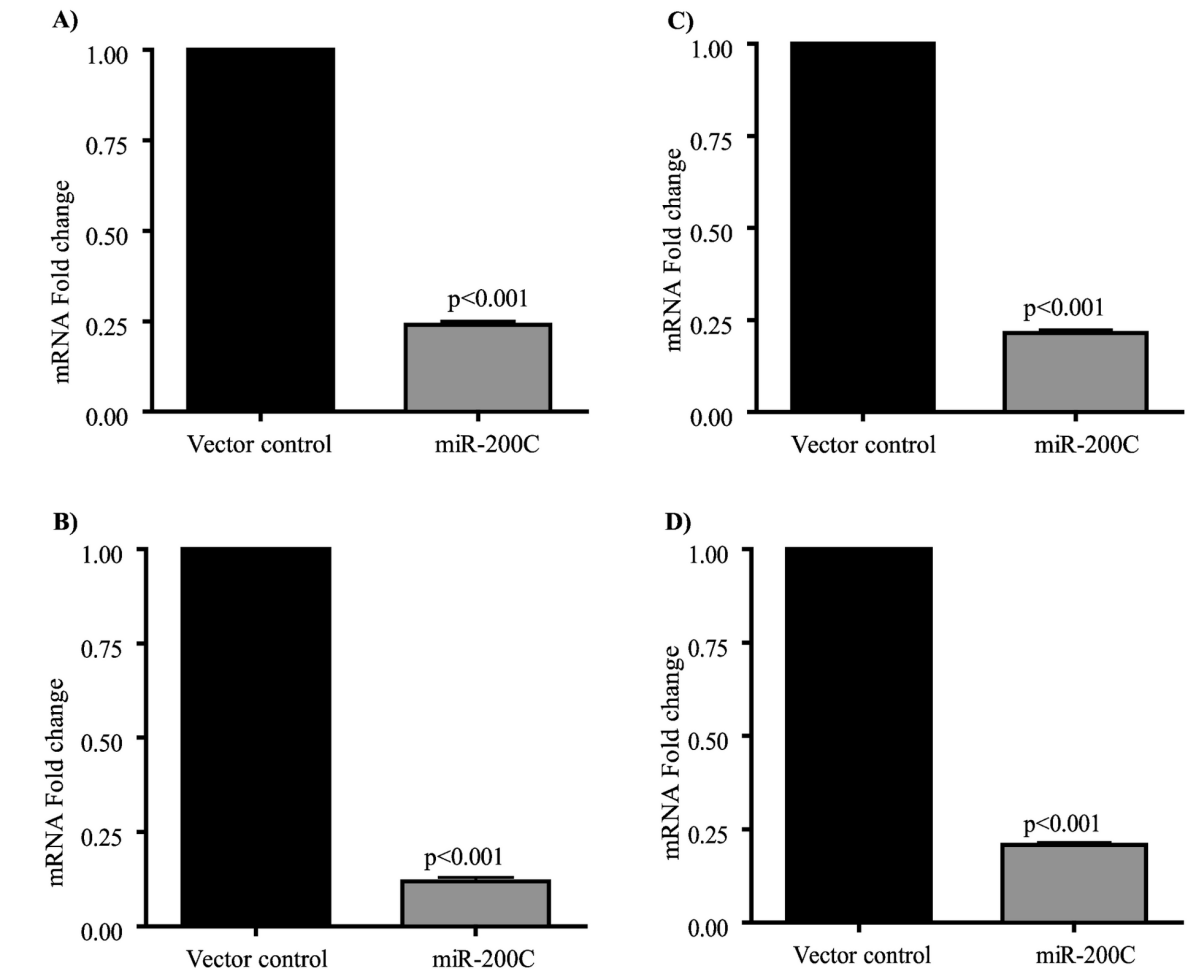
miR-200c targets transcript levels of MUC4 and MUC16 in S2.028 and T3M-4 cells. qRT-PCR analysis of MUC4 (A and B) and MUC16 (C and D) were carried out in vector control and miR-200c transfected S2.028 (A and C) and T3M-4 cells (B and D). The relative expression of MUC4 and MUC16 was evaluated by the 2^-ΔΔCt^ method using GAPDH as an internal control. The miR-200c expressing cells (S2.028 and T3M-4) showed significantly reduced levels of MUC4 mRNAs compared to vector control cells (A and B). Expression of MUC16 transcript was reduced in miR-200c expressing S2.028 and T3M-4 cells (C and D). All measurements were carried out in triplicate. The fold increase in transcript levels over vector control were expressed as Mean ± S.D. A p-value less than 0.05 was considered to be statistically significant.

We also observed a significant reduction of both MUC4 and MUC16 mRNA levels in miR200c expressing cell lines. MUC4 transcripts were reduced 4.18 and 8.50 fold in S2.028-miR200c and T3M-4-miR200c respectively (Figure 4A and 4B) compared to control cells. MUC16 transcripts were reduced 4.68 and 4.82 fold in S2.028-miR200c and T3M-4-miR200c respectively, compared to control cells (Figure 4C and 4D). These findings indicate that miR-200c regulates expression of oncogenic MUC4 and MUC16 transcription and translation.

### Prediction of miR-200c binding sequences on MUC4 and MUC16

**Figure 5:**
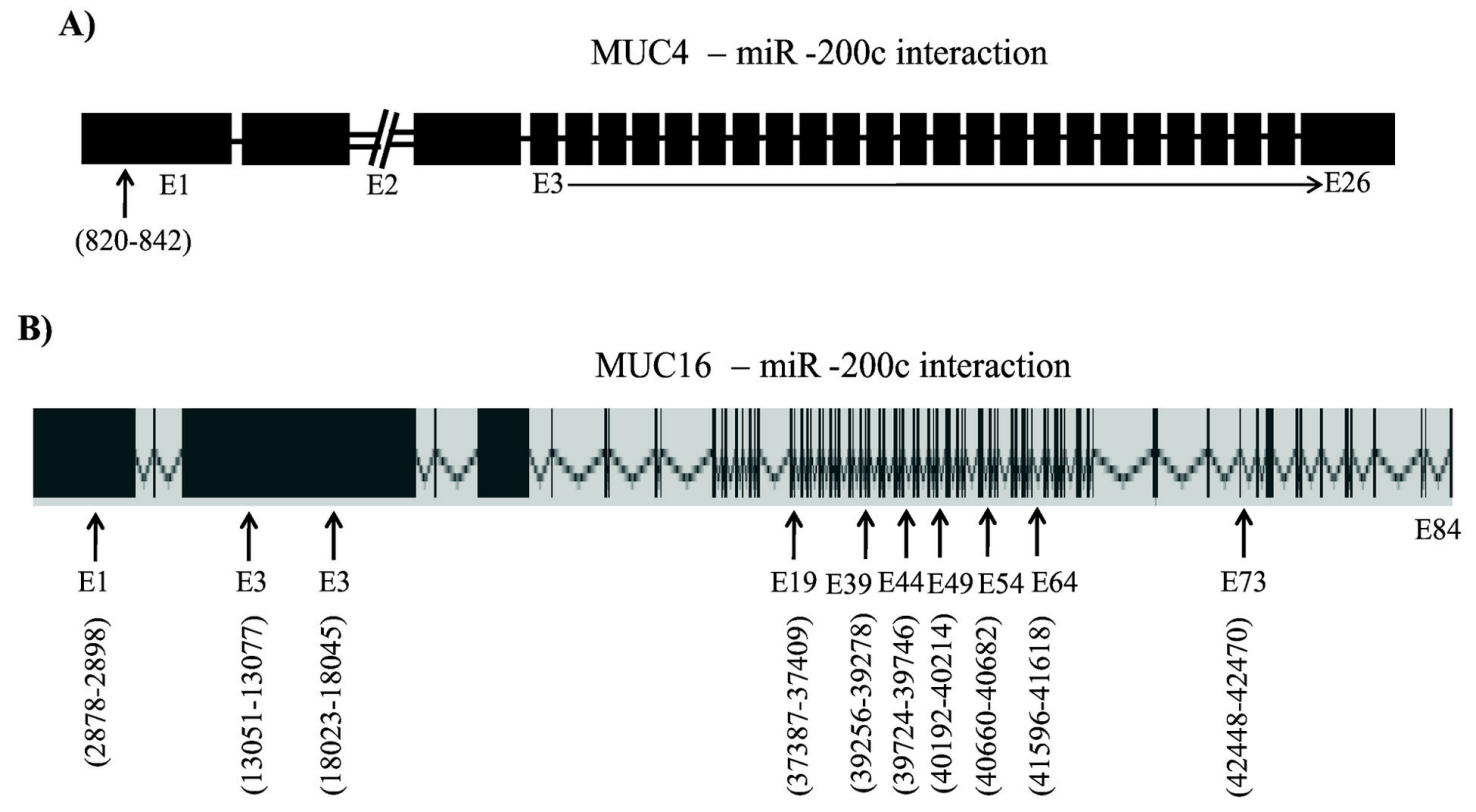
Prediction of miR-200c interaction sites in MUC4 and MUC16 genes. Possible miR-200c targeting regions in MUC4 and MUC16 were identified by using the RegRNA MicroRNA target prediction web server (http://regrna.mbc.nctu.edu.tw/index1.php). A, RegRNA miRNA target prediction shows that miR-200c binds between base pairs 820-842 in the first exon of MUC4. B, In MUC16 mRNA, the miR-200c is predicted to bind nine different exons including E1, E3, E19, E39, E44, E49, E54, E64 and E73. The numbers indicate the region of mRNAs that interact with miR-200c.

Potential miR-200c targeting regions in the MUC4 and MUC16 transcripts were identified by using the RegRNA MicroRNA target prediction web server (http://regrna.mbc.nctu.edu.tw/index1.php). RegRNA miRNA target prediction identified high probability for miR-200c binding to coding sequences of MUC4 between base pairs 820-842 (Exon-1) (Figure 5A) (Figure S3) and ten different regions including nine different exons within MUC16 mRNA sequence (Figure 5B) (Figure S3). These data led us to determine if miR-200c could directly target MUC4 and MUC16 transcripts within the translated regions.

### miR-200c directly targets the coding sequences of MUC4 and MUC16

**Figure 6:**
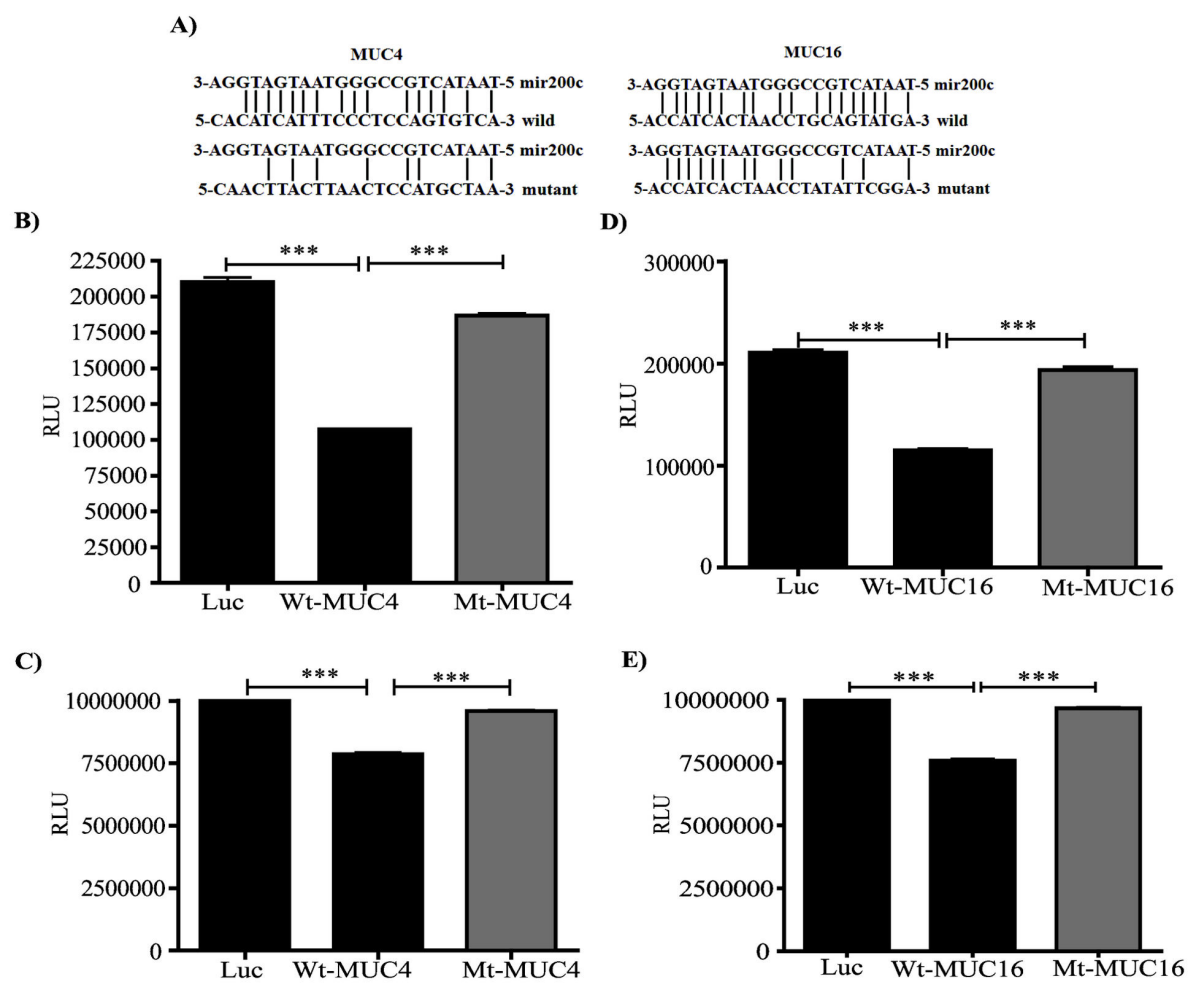
Luciferase assay showing that miR-200c regulates MUC4 and MUC16 expression in S2.028 and T3M-4 cells. (A) Wild type and mutated sequences of MUC4 and MUC16 were cloned into the pMIR-Luciferase vector (pMIR-MUC4 wt and MUC16 wt) and (pMIR-MUC4 mt and MUC16 mt) respectively. Vectors expressing pMIR-Luc, pMIR-MUC4 wt, pMIR-MUC16 wt, pMIR-MUC4 mt and pMIR-MUC16 mt were transfected into S2.028miR-200c (B and D) and T3M-4miR-200c cells (C and E) and luciferase activity was quantified 48 h after transfection. The results were expressed as relative luciferase activity (Mean ± SEM of three independent determinations). The p value was determined using Student’s t-test (*** p<0.0005).

We evaluated binding of miR-200c to the putative target sequence by cloning the regions containing human MUC4 and MUC16 coding sequences into the pMIR-Luciferase reporter vector (pMIR-MUC4 and MUC16 wt). Negative controls were produced in which the pMIR-MUC4 and MUC16 sequences were mutated at the putative binding sites for miR-200c and the mucins (pMIR-MUC4 and MUC16 mt) (Figure 6A). S2.028 and T3M-4/miR-200c cells were transiently transfected with plasmid DNAs encoding vector alone, wild type MUC4 and MUC16 sequences in the vector, and mutant type MUC4 and MUC16 sequences in the vector. We observed that luciferase reporter activity was significantly suppressed for both MUC4 and MUC16 in S2.028 and T3M-4 miR-200c cells transfected with wild type sequence vectors as compared to vector control transfected cells (***p<0.0001, MUC4-wt, MUC16-wt vs vector control) (Figure 6B-E). However, cells transfected with the mutant sequence containing vectors showed significantly increased luciferase reporter activity (***p<0.0001, MUC4-wt vs MUC4-mt; MUC16-wt vs MUC16-mt), (Figure 6B-E).

## Discussion

In the present study, we demonstrate for the first time that a mirR-200c targets high molecular weight mucin glycoproteins by targeting their coding sequence for degradation or translational inhibition. Specifically, miR-200c targets MUC4 and MUC16 exon sequences to regulate mRNA and protein levels for each of these mucins. We have shown that miR-200c is differentially expressed in pancreatic cancer cells. Yu et al observed a significantly higher survival rate in pancreatic cancer patients with higher levels of miR-200c compared to those with lower levels of miR-200c [10]. Previous work has also shown that pancreatic cancer cells overexpressing miR-200c exhibit reduced invasion and increased levels of E-cadherin mRNA, suggesting that miR-200c plays an inhibitory role in pancreatic cancer growth and invasion [10]. Overexpression of miR-200c increased the rate of proliferation in SUIT-2, PANC-1 and KP-3 pancreatic cancer cells [10], however, we observed reduced cell proliferation of S2.028 miR-200c expressing cells, suggesting that the cell growth properties differentially regulated by miR-200c are context dependent. MiR-200c has been described as a powerful master regulator of epithelial-to-mesenchymal transition in breast and ovarian cancer cells through altering the expression of ZEB1 and E-cadherin by binding to 3’ UTRs and driving degradation or blocking translation of the target mRNA [11-14]. Restoration of miR-200c expression in highly aggressive breast and ovarian cancer cells significantly reduced invasion, migration and drug resistance of these tumor types [30,31]. These reports suggest that introduction of miR-200c into cancer cells could reverse tumor progression and provide a novel treatment strategy.

A majority of reports show that miRNAs target the non-coding 3’UTR of mRNAs; however, recent reports have shown that miRNAs can also target coding sequences of mammalian genes. We report here that miR-200c targets exon coding sequences in transcripts of high molecular weight mucin glycoproteins MUC4 and MUC16 and reduces mRNA and protein expression. Similarly, Huang et al, reported that miR-181a targets a large number of zinc finger genes by binding to their coding sequences [32] and Duursma et al reported that miR-148 targets human DNA methyltransferase 3b (DNMT3b) amino acid coding regions [33]. Let-7 miRNA targets the coding region of miRNA processing enzyme, Dicer [34]. Murine Nanog, Oct4 and Sox2 genes contain numerous binding sites in exon coding sequences for naturally occurring miRNAs [35]. These reports suggest that miRNAs target specific gene families within coding regions.

Regulation of MUC4 and MUC16 by miR-200c may be through effects on either transcription or translation. In both cells lines studied here, MUC16 protein levels were reduced to a greater extent than MUC4. This increased targeting of MUC16 by miR-200c may be due to the presence of ten different miR-200c binding regions in the MUC16 mRNA compared to the single binding region in the MUC4 coding sequence, raising the possibility that the number of targeting sites in a transcript influences the sensitivity of that transcript to miRNA regulation.

Recently, Ponnusamy et al reported that overexpression of MUC4 results in epithelial to mesenchymal transition through down regulation of E-cadherin and increased cancer cell migration and invasion in ovarian cancer cells [36]. These results are consistent with our study, which shows that overexpression of the mesenchymal to epithelial promoting miR-200c down regulates ZEB1 and MUC4 in pancreatic cancer cells.

MUC16 is highly overexpressed in ovarian cancer and moderately overexpressed in pancreatic cancer, but the role of MUC16 in pancreatic cancer progression has not been extensively studied. The significantly reduced level of MUC16 observed in miR-200c overexpressing cells, along with the well documented role of miR-200c in multiple stages of cancer progression suggests that MUC16 may have a role in pancreatic cancer cell growth and metastasis. These studies indicate that up-regulation of mucins and downregulation of miR-200c enhance the cancer cell malignant properties and thereby induce pancreatic cancer growth and metastasis.

Several studies have shown that aberrant and increased expression of MUC4 and MUC16 occurs during pancreatic cancer progression to metastasis [17-19,25]. Recently, Mohr et al showed miR-200c expression was reduced in primary pancreatic tumor tissues as compared to some metastatic sites [37]. These results generally support the hypothesis that fluctuations in miR-200c contribute to regulation of expression of MUC4 and MUC16 during pancreatic tumor progression and metastases.

In conclusion, our study presents the first evidence that MUC4 and MUC16 are regulated at the post-transcriptional level by a miRNA, miR-200c, which directly targets MUC4 and MUC16 within their amino acid coding regions. These results indicate that miR-200c may have a potential inhibitory role in pancreatic cancer growth and metastasis and additional studies are required to delineate the regulatory relationship between membrane bound mucins and miR-200c.

## Supporting Information

Figure S1 **qRT-PCR analysis of ZEB1.** Overexpression of miR-200c significantly reduced the expression of ZEB1 in both (A) S2.028 and (B)T3M-4 cells. The fold increase in transcript levels over vector control were expressed as Mean ± S.D. A p-value of less than 0.05 considered to be statistically significant.
(EPS)

Figure S2 **Cell proliferation assay.** S2.028 (A) and T3M-4 (B) (miR-200c and vector control) cells proliferation at different time points were measured by methylene blue assay. Two Way ANOVA was used to obtain statistical significance (***, p<0.001; ns, non-significant).
(EPS)

Figure S3 **Prediction of miR-200c binding sites in mucins.** Potential miR-200c binding sites in the membrane bound mucins (MUC4 and MUC16) transcripts were identified by using the RegRNA MicroRNA target predictor web server.
(EPS)

Table S1 **List of oligonucleotides used for miR-200c expression and luciferase assay system.**
(DOCX)
