# Supplementary material for: MicroRNA-200c Modulates the Expression of MUC4 and MUC16 by Directly Targeting Their Coding Sequences in Human Pancreatic Cancer
Source: PLoS One. 2013 Oct 25;8(10):e73356. doi: 10.1371/journal.pone.0073356 (PMC3808362; doi:10.1371/journal.pone.0073356)
Supplement: Table S1 — List of oligonucleotides used for miR-200c expression and luciferase assay system. (DOCX) [file pone.0073356.s004.docx]

**Table S1**

| **miR-200c oligos** |
| --- |
| CCCTCGTCTTACCCAGCAGTGTTTGGGTGCGGTTGGGAGTCTCTAATACTGCCGGGTAATGATGGAGG |
|  |
| **MUC4-wild oligos** |
| Forward  5-CTAGTCAGATGAAATGACCACATCATTTCCCTCCAGTGTCACCAACACACTCATGA-3 |
| Reverse  5-AGCTTCATGAGTGTGTTGGTGACACTGGAGGGAAATGATGTGGTCATTTCATCTGA-3 |
| **MUC4-mutated oligos** |
| Forward  5-CTAGTCAGATGAAATGACCAACTTACTTAACTCCATGCTAACCAACACACTCATGA-3 |
| Reverse  5-AGCTTCATGAGTGTGTTGGTTAGCATGGAGTTAAGTAAGTTGGTCATTTCATCTGA-3 |
|  |
| **MUC16-wild oligos** |
| Forward  5-CTAGTCACCCTCAATTTTACCATCACTAACCTGCAGTATGAGGAGGACATGCGTCA-3 |
| Reverse  5-AGCTTGACGCATGTCCTCCTCATACTGCAGGTTAGTGATGGTAAAATTGAGGGTGA-3 |
| **MUC16- mutated oligos** |
| Forward  5-CTAGTCACCCTCAATTTTACCATCACTAACCTATATTCGGAGGAGGACATGCGTCA-3 |
| Reverse  5-AGCTTGACGCATGTCCTCCTCCGAATATAGGTTAGTGATGGTAAAATTGAGGGTGA-3 |
